# Parent recommendations to support physical activity for families with young children: Results of interviews in deprived and affluent communities in South Wales (United Kingdom)

**DOI:** 10.1111/hex.13020

**Published:** 2020-01-02

**Authors:** Ashrafunnesa Khanom, Bridie Angela Evans, Rebecca Lynch, Emily Marchant, Rebecca A. Hill, Kelly Morgan, Frances Rapport, Ronan A. Lyons, Sinead Brophy

**Affiliations:** ^1^ Swansea University Swansea UK; ^2^ National Centre for Mental Health Cardiff University Cardiff UK; ^3^ Hywel Dda University Health Board Wales UK; ^4^ Present address: Macquarie University Macquarie Park NSW Australia

**Keywords:** family activity, interventions, interview, physical activity, young children

## Abstract

**Background:**

Physical inactivity is the fourth leading cause of mortality worldwide. Early childhood is a critical period when healthy behaviours can be instilled for a future active lifestyle. We explored community, societal and environmental factors affecting child and family physical activity and sought parent recommendations to support physical activity in families with young children.

**Methods:**

We interviewed 61 parents expecting a child or with a baby ≤12 months (35 mother and father paired interviews and 26 interviews with mothers only). We purposively sampled families for neighbourhood deprivation status (Townsend Index; 26 affluent; 35 deprived). We conducted thematic analysis of interview transcripts using Bronfenbrenner's socio‐ecological framework to guide interpretation.

**Results:**

We identified four themes: work family‐life balance; spaces for activity; beliefs and attitudes; and physical activity facilitators. We found that parents from deprived neighbourhoods were more likely to be underactive because of a complex web of community, social and personal factors which reduced motivation and hindered opportunity for physical activity. To increase knowledge and opportunity, respondents suggested ‘help not tell’ messages covering ‘why’, ‘how’ and ‘where’ information about physical activity, and using physical activity to support community engagement and social interaction.

**Conclusions:**

Recommendations from parents highlight effective communication about the importance of early child and family physical activity and improved community access to safe facilities and opportunities. Both parents need to be engaged in designing interventions to support greater physical activity and healthy behaviours which are relevant and achievable in individuals’ lives.

## 1 BACKGROUND

Physical inactivity is the fourth leading cause of mortality worldwide.^1^ Greater urbanization and development of modern technology contribute to inactivity and its associated physical and psychological health problems.^2^, ^3^ Inactivity and sedentary behaviour start from an early age and are linked to weight gain through childhood.^4^, ^5^ Studies have identified early metabolic markers for high cholesterol, blood pressure and abnormal glucose metabolism in overweight children as young as five^6^ and early vascular lesions in overweight children as young as three.^7^

Many pre‐schoolers (2‐6 years) fail to complete the minimum daily 60 minutes of moderate‐to‐vigorous physical activity recommended for young children.^8^ Targeting interventions at early years (0‐8 years) could support formation and maintenance of future health behaviours^1^ when physical activity can be nurtured to sustain active lifestyles.^9^, ^10^ The Department of Health in the United Kingdom has produced physical activity guidelines for children from birth to 5 years of age to encourage activity in early childhood.^11^

Increasing whole‐family physical activity could potentially reduce childhood obesity and improve their overall health. Poor motor development in infancy and early childhood is associated with reduced physical activity in older children but can be improved through parent‐facilitated physical activity at an early age.^12^, ^13^ Children's activity rates increase if one parent is active and are highest if both parents are active.^14^ Children with active parents are more likely to maintain activity levels through childhood.^15^, ^16^, ^17^, ^18^ However, adults with dependent young children appear to be less physically active compared to those without children.^19^, ^20^ Moreover, parents^21^ and adults in general^22^, ^23^ undervalue the importance of physical activity as a means to encouraging energy expenditure and reduce weight gain.^24^, ^25^

Interventions which focus on individuals and their families can overlook social, economic and environmental barriers to undertaking physical activity; which individuals often have little control over and can prevent them maintaining positive health behaviours.^26^, ^27^, ^28^, ^29^, ^30^, ^31^ Young mothers from disadvantaged backgrounds are less likely to participate in regular physical activity.^32^ Children from families in lower socio‐economic groups have lower levels of physical activity^33^, ^34^ and higher body mass index.^35^ Studies looking at parent‐reported barriers report lack of time,^36^, ^37^ the cost of being active,^36^, ^37^ facilities within the home 36, 38 and outside.^39^

**Figure 1:**
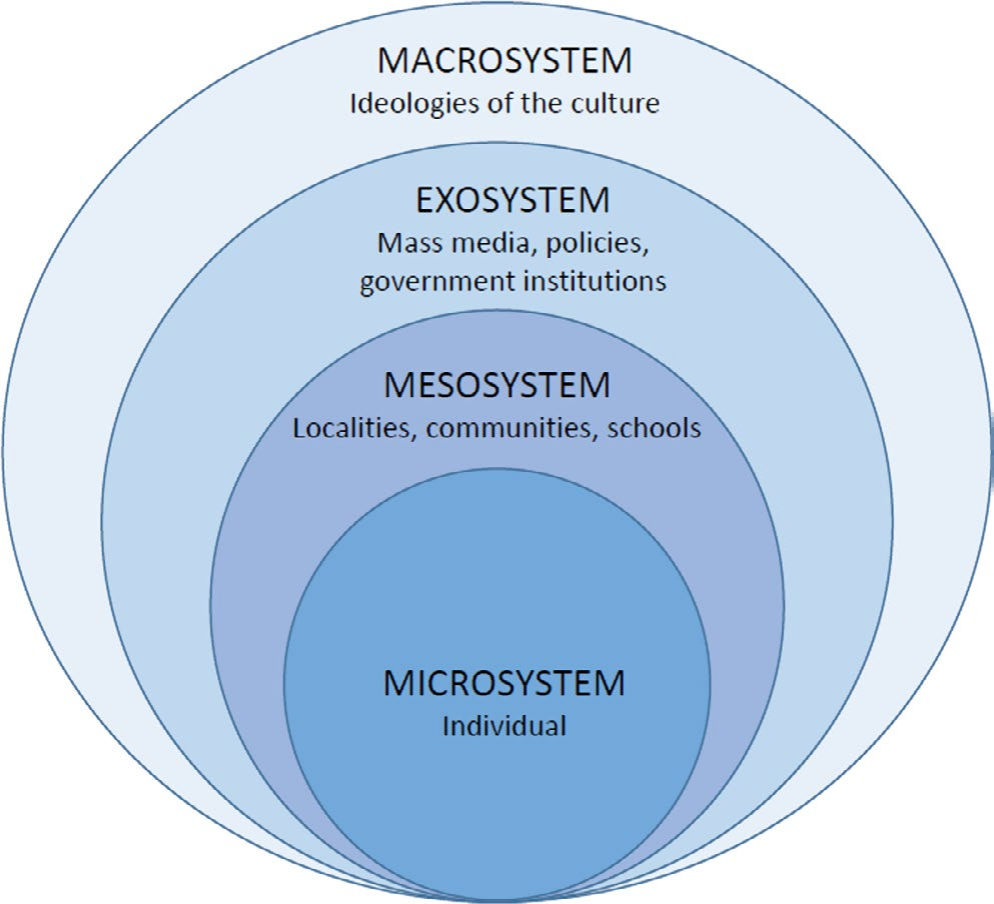
Bronfenbrenner's socio‐ecological model. Source: Bronfenbrenner^40^

One way to understand how context influences parent and child health behaviours is through Bronfenbrenner's socio‐ecological model.^31^, ^40^, ^41^ This identifies four levels of influence on a child's development: the individual's immediate physical and social environment such as home and family (microsystem); the wider environment such as school (mesosystems); broader social, political and economic conditions (exosystem). These all interact to affect the beliefs and attitudes of wider society (macrosystems; see Figure 1).

This study explores the attitudes of parents across different socio‐economic groups towards physical activity and the opportunities available to them. We use Bronfenbrenner's socio‐ecological framework to address the reported evidence gap in ways to enable more physically active children.^42^, ^43^ In this paper, we report the views of expectant parents and families with a baby ≤12 months concerning community, societal and environmental factors affecting family physical inactivity, as this can be a time when family activity levels can decline. We also present parents’ recommendations for interventions to support physical activity in families with young children.

## 2 METHODS

### 2.1 Setting

Parents in this qualitative study were already taking part in an existing birth cohort study ‘Growing Up in Wales: Environments for Healthy Living’^44^ which examined the impact of environmental influences during gestation and post‐birth on health outcomes using data linkage of routine, anonymized medical records.^45^ Participants were recruited for the cohort study when they attended maternity appointments in hospitals and clinics. Exclusion criteria were pregnancies under the age of 16, incomplete pregnancies and mothers with serious health problems such as cancer. A detailed description of study participants who were eligible to take part in the birth cohort study has been provided elsewhere.^44^

### 2.2 Qualitative study: recruitment and data collection

We purposively sampled parents from the birth cohort sample of parents according to neighbourhood deprivation using the Townsend Deprivation Index.^46^ AK contacted expectant mothers or mothers with infants aged ≤12 months, face to face at antenatal clinics or by telephone. AK made arrangements to interview those who agreed to take part. Interviews took place in participants’ homes except for two conducted at participants’ workplace (personal preference). A semi‐structured interview schedule was used for interviews (Table 1). Questions concerned parents’ knowledge and views about living a healthy lifestyle; how this influenced their current choices for family‐based physical activity; barriers and facilitators to physical activity; and recommendations to facilitate family physical activity. Informed consent was obtained before undertaking and recording the interviews, which were all carried out by AK. Interviews were conducted in English, Bengali and Urdu depending on which language was preferred by respondents. Parents who spoke Urdu and Bengali were included in the sample as the researcher AK was fluent in both these languages. The interviews ranged from 30 to 60 minutes.

**Table 1:** Interview schedule for physical activity

Can you describe your weekly levels of physical activity (individually and as a family)?
Does anything pose a barrier to your family taking part in physical activity?
Local environment and location of facilities?
Beliefs, attitude, culture?
During your childhood how physically active were you?
Can you identify the best way to inform or advise parents about the importance of physical activity?
What sort of service or support do you look to health professionals for?
What sort of families do you think will/should use or listen to advice on taking part in physical activity?
Do you know of anything being done in your local area to promote physical activity?
Do you have any ideas on ways to improve your local area to encourage family physical activity?

Recruitment and data collection happened in parallel. Initially, the intention was to interview mothers only. However, fathers were present in two early interviews and it was recognized that this broadened the discourse and enabled data capture of the whole‐family perspective, and useful detail of the dynamics between parents. Subsequently, AK sought to recruit fathers also. During the paired interviews, AK encouraged both participants to equally contribute to the discussion by rephrasing and directing questions to both participants.

### 2.3 Data analysis

An inductive thematic data analysis approach was used^47^ where theoretical perspectives are informed by the interpretation of raw data. AK, RL and EM independently reviewed anonymized interview transcripts, working with the first 20 completed interviews to systematically code the data to draw out themes and categories to illustrate emerging concepts. Through discussion, these themes and categories were further refined and clustered into codes and sub‐codes concerning individual/family, community, environmental and societal level, using the socio‐ecological model as a framework^40^ (see Figure 1). These formed a codebook which was used by AK to code the remaining transcripts. Findings were discussed in four paired analysis sessions, to ensure robustness and internal validity.^47^, ^48^ A senior qualitative researcher (FR) was also available throughout to challenge and critique analytic outputs and themes as they emerged.

### 2.4 Reporting

We report results according to themes identified in the data. We selected quotations to be representative of respondents’ comments unless otherwise stated. Quotations are identified by respondent family identification number and whether they are a mother or father. Further information such as deprivation status of where they live and age and number of children can be found in Table 2.

**Table 2:** Identifiers for study quotes

Respondent family ID	Neighbourhood details	Family details
1 Mother	Deprived	Child aged three and child aged 4 months
2 Mother	Deprived	Child aged 4 months
6 Mother	Affluent	Child aged eight, six and two
7 Mother	Deprived	Child aged six, four and expecting child
12 Mother	Affluent	Child aged 6 months
13 Mother	Deprived	Child aged eight and child aged 6 months
14 Mother	Deprived	Child aged six and child aged 9 months
17 Mother	Deprived	Child aged one
18 Mother	Deprived	Child aged three and child aged 9 months
19 Mother	Deprived	Child aged 9 months
21 Father	Deprived	Child aged four and expecting child
21 Mother	Deprived	Child aged four and expecting child
22 Mother	Deprived	Child aged two and expecting child
23 Father	Affluent	Child aged two and expecting child
25 Mother	Affluent	Child aged two and expecting child
26 Mother	Affluent	Expecting child
28 Father	Deprived	Expecting child
35 Father	Affluent	Child aged four and expecting child
41 Mother	Deprived	Child aged eight and child aged 3 months
49 Mother	Affluent	Child aged four and one
55 Mother	Deprived	Child aged five, child aged 12 months and expecting child
60 Father	Deprived	Child aged 19 months and child aged 8 months

## 3 RESULTS

We conducted 61 interviews from a potential total cohort of 819 families from the Growing Up in Wales study with parents: 35 with both parents and 27 with mothers only; 26 families from affluent neighbourhoods; and 35 families from deprived neighbourhoods. Respondent characteristics are shown in Table 3. Four interviews were conducted with mothers only in the participant's mother language (Bengali [3] and Urdu [1]) and were translated and transcribed by the researcher (AK). The remaining transcripts were transcribed by an independent transcriber. All transcripts were cross‐checked with notes taken during the interviews to ensure data integrity. We identified four themes: work family‐life balance; spaces for activity; beliefs and attitudes; and physical activity facilitators.

**Table 3:** Socio‐demographic data of study participants

Socio‐demographic characteristics of participants	
Number of interviews conducted	61
Parents (n)	
Mother	61
Father	35

	Mother	Father
Average age in years (age range of all parents in family)	30 (20‐42)	35 (21‐52)
Occupation of all parents (n)		
Non‐manual	16	27
Manual	17	26
Student	5	3
Not in employment	11	4
Homemaker	12	1
Education of all parents (n)		
Higher degree	16	12
First degree	15	13
Diplomas in higher education	6	3
A/AS levels	4	3
O/GCSE levels	14	14
Other	2	12
None	4	3
Unknown	0	1
Ethnicity of all parents (n)		
White (European)	49	52
Welsh	47	51
Romanian	1	0
Polish	1	1
Ethnic minority	12	9
Bangladeshi	4	4
African	2	1
Middle Eastern	2	2
South East Asian	2	1
Pakistani	2	1
Socio‐economic status % (n)		
Affluent neighbourhood	43% (26)	
Deprived neighbourhood	57% (35)	
Number of children % (n)		
Expectant mother with no child	11% (7)	
Expectant mother > 1 child	48% (29)	
Mother with child ≤ 12 months of age	41% (25)	
Average number of children per family (range = n)	1.7 (1‐5)	
Average age of children (age range)	4.75 y (1 months‐18 y)	
Number of children in each age category(n)		
<1 y	20	
1‐1.9 y	12	
2‐2.9 y	13	
3‐5 y	20	
>5 y	39	

### 3.1 Work family‐life balance

Respondents reported that busy work schedules, irregular work patterns and lack of time reduced opportunities for family physical activity. Many parents expressed a keen desire to be active as a family unit and often sought support from each other to facilitate family activities, recognizing value for whole‐family health and well‐being and also the opportunity to maintain and strengthen family bonds. However, work patterns resulted in extended periods of time when one parent was alone with one or more young child. Often, one parent had to stay home to wait for their partner to return. This was especially evident for mothers living in deprived areas. Repeated and prolonged periods alone caring for their children also appeared to be a bigger burden for mothers. They reported that it resulted in less time playing and being active with their children. Sometimes this was because several adults were needed to make an activity possible. Managing and supervising several very young children, handling pushchairs and other equipment, was very difficult for one parent. Public facilities often required more than one adult. Parents reported feeling isolated and less willing or able to take their children out on their own or when they had household tasks to undertake—unless it was shopping trips.

The only thing with that [trying to go swimming as a family] if you’ve got children under a certain age you need to be a one to one. ..Three kids…a slight problem, you need three adults (55: Mother)

Being alone at home also eroded motivation to be active and to play. Arranging activities as a sole parent could be less fun as well as harder to manage.

I’d like to move towards doing more things as a family like family bike rides and so it would be all of us rather than [on our own]…we’ll spend longer, longer outside. If it’s just me and X [daughter] then a lot of the time we’ll have something specific to do rather than too much playing (22: Mother)

Many fathers, from both types of communities, voiced dissatisfaction with their employment patterns because work hours reduced the time they could spend with their children and limited the possibility of regular physical activity.

Because [of] my teaching, you’re limited to the holiday doing things and as soon as I’m home I just want to do stuff with her (23: Father)

The effect of employment patterns was more acute when one or both parents were working shifts or overtime for additional income, which was more common for those in deprived neighbourhoods. Having regular work hours enabled families to schedule activity during the week and at weekends.

My job is different hours virtually every day… at the moment, [my partner] had the kids all day, she’d go to work and then I’ve got them for about two hours before they go to bed… but I don’t know if the government could do anything to sort of allow us time maybe off work and things like that, whether we could spend more time with the kids (60: Father)

Access to personal transport was associated with employment for many families. A family needed two vehicles, if one was used by the parent travelling to work and the parent at home had one too. Families needed a larger income to run one or two cars. Families with access to a car had more choice and opportunity to pursue physical activities for children. Access to facilities often required access to transport and added to the overall cost of attending such facilities. Income was a factor in choosing physical activities, with some respondents reporting that some activity venues were too expensive to visit regularly.

Well when we went down the beach [by taxi] we ended up walking back into town. But then when we go out we have a taxi home because the double buggy steers like a tank and it’s heavy because we’ve got the two of them and he’s no lightweight, so it’s easier to get a taxi home,…we’ve always got at least £10 to manage to get a taxi home so [adds to the cost of being active as a family] (18: Mother)

### 3.2 Spaces for activity

The home was identified as an important space for enabling or restricting physical activity. In small homes with limited floor area, children played in confined indoor spaces or potentially unsafe areas such as steps in front of the house when there was no garden. These children were reportedly more likely to encounter accidental injury around the home.

[My daughter aged 2/3 has hurt herself playing in here] diving off the settee and bouncing off the fire [guard] with her head (18: Mother)

A number of homes in the deprived areas had steps to the main door. These created extra barriers to families going out to be active together, exacerbated because the parent had to manage alone when the partner was working. One mother, pregnant with her third child, said she felt imprisoned in her house because she could not leave with the children on her own.

We’ve got a double buggy and I only go out when their father’s off… …unless there’s two people I can’t get the pram down the steps…but if we moved to somewhere with less steps we would probably go out a lot more (18: Mother)

Other participants living in deprived areas relied on friends and family networks to help them access spaces for safe play:

….our friends have just moved down into the new [X] Estate so we go down to see them and then we spend all day running round the garden (18: Mother)

In contrast, children living in mostly affluent neighbourhoods were observed by the researcher to have access to more floor space within the home that was safe for play and physical activity. These homes also tended to have bigger gardens, which were better equipped for the children.

…. we’ve got a massive trampoline that they’re out on every night, you know, we’ve got a big garden that they play in that, you know, they use their bikes. So I would say the children are very active, even on days when we stay at home (6: Mother)

In addition, they often had access to well‐maintained facilities such as parks and local amenities near their homes which enabled them to be active as a family.

Yeah, but we’re lucky I guess for exercise ‘cause the park’s at the back of the house, so we can just walk in there, good fresh air, great environment, but I think if it wasn’t there, if it was more difficult to get to it I think we’d be struggling (35: Father)

Overall, they were less reliant on partner support to facilitate daily activities. They were also likely to have access to their own personal transport which offered greater freedom of choice in the type of activities that were offered to children.

I’m out at work two days a week, and the rest of the week we tend to go to baby groups in the mornings, we go to the library and we go swimming, we do sign language and music (12: Mother)

#### 3.2.1 Safety and accessibility

Many respondents talked about their safety in outdoor spaces, in addition to the physical risks in vandalized or dirty public parks. Respondents who lived in deprived areas were unhappy with the condition of their local parks. They said the facilities were damaged and the ground was littered with needles, scrap metal, broken glass or fouled by dogs which created safety concerns, sometimes so badly, that the parks were unusable.

I took my little boy up there on the bike and I couldn’t leave him on it, if he fell off he’d land in glass, it’s dreadful over there [local park] (14: Mother)

We don’t go to the parks round here… Because of heroin addicts… you can find needles (21: Father)

A number of people perceived risks in areas where there were few other people, or people they didn't trust. In parks and on cycle paths and walking routes, respondents said they felt anxious for their safety and the safety of their children because these facilities were little used, overgrown or poorly designed. These concerns were shared by parents in both types of communities.

…there’s a cycle path which runs sort of from about eight miles up that direction … but as if a girl would go there on her own… Too scary! (26: Mother)

Respondents also asked for investment in their local built environment to repair and expand existing facilities and ensure public spaces were safe and accessible for family activities. Better cycle routes and safer local parks were frequently requested as easy ways to support people's potential interest.

…we like going out on our bikes and there doesn’t seem to be an awful lot of cycle paths just here… Yeah some safer places to go on a bike (17: Mother)

Some participants identified the need for facilities for older children and teenagers because of perceptions that they contributed to local parks being unsafe and inaccessible for young families. These comments were particularly prominent among respondents living in deprived areas. The presence of local, safe cycle paths was reported to be a strong motivator for families to engage in physical activity and also allowed free travel in their communities.

…if the cycle ways were safer for us to use as a family then we’d be able to get around the city, and up to school and back and into town, and, on our bikes wouldn’t we? (49: Mother)

Access to organized activities and facilities locally was also reported to be variable. Some parents commented that there were few clubs for older children in their local area and those that were available were often oversubscribed, further limiting opportunities.

No, there’s nothing for them to do, no, nothing at all. Well, I think there’s a football pitch all the way up there somewhere, but there’s a lot of gypsies up there, actually, so I don’t know, to be honest…I don’t think there’s any clubs round here at all, nothing like that, which I think they [children] need (41: Mother)

the school, they have like Fit Club and all this, but it’s full. I always ask them when they have space for him, and they always say they will make sure (13: Mother)

Access to safe walking routes was also a concern for parents from both types of community. They were hesitant about walking their children to school, even though this daily routine provided a ready‐made opportunity for physical activity. They said the walk was too long, taking too much time and beyond their children's ability; traffic was too fast, and it was dangerous to cross the road with a pram:

… even the school run for me is taking your life in your hands, you know, because I don’t get on a pavement until I’m almost at school and it’s a single lane, wooded lane that I’m walking around with a buggy and two other children to look after and it, that walk freaks me out a bit I must admit. So I tend to try and avoid that (6: Mother)

### 3.3 Beliefs and attitudes

Parents appeared to understand the importance of physical activity, for their children and also for their family. Although some mentioned it in a health context, activity was more often discussed as a time for being with other people. Some parents said they enjoyed cycling as a family. While being active benefited everyone's health, doing something together appeared to be just as important for strengthening the family unit.

…it’s a bonding time with you and your family and it’s keeping healthy together, you’re supporting each other’ (19: Mother)

Community activities were seen as a positive way for adults and children of all ages to be together. By contrast, inactivity was associated with personal and community problems.

I think they need to give something for the older children to do. And I know we bang on about youth clubs and things like that, but they’re sitting there just making a menace and then they graffiti the new park things and you think, well it’s sad because this is for the younger children, not for you (7: Mother)

Attitudes to physical activity were affected by how much it was considered a normal aspect of life. Individuals who had always been active perceived it as part of their everyday habits, routinely walking or cycling and then adding other activities to that schedule.

I used to do triathlon…as a young child, it would be ballet on a Monday, swimming on a Tuesday… we do lots of walking as well [as a family], but we don’t think of that as exercise do we? (25: Mother)

Other respondents, whose routine provided less opportunity for being active, perceived activity as an additional thing to arrange, often involving inconvenience or difficulty and they reduced their expectations accordingly.

No I don’t mind, I could walk but… you know, I don’t think my daughter [three years old] can walk for 20 minutes, you know, it’s too much for her (1: Mother)

Parents’ attitudes towards physical activity also appeared to be strongly influenced by the quality of the environment. Some respondents associated beaches and countryside with an active lifestyle and consciously chose to live in these areas so they could follow an active way of life. The opportunity to move to such areas was primarily evident among families with higher income, suggesting inequities in choice associated with socio‐economic status:

I think that is one of the big factors why we moved here as well ‘cause we appreciate healthy lifestyle, we wanted to be by the sea, we wanted the parks (35: Mother).

### 3.4 Physical activity facilitators

Parents asked for information to encourage physical activity, with messages applicable to a family which explained why physical activity was valuable, how to do it and where to access local opportunities. Supportive and relevant messages, ‘helping’ not ‘telling’, were more likely to encourage people in place of a didactic approach which risked alienating those who found the advice hard to follow through.

You should do this, you should do that, you have to do this’. It’s great in theory, but unless you can provide actually a way and a means of doing that practically, on a day‐to‐day basis, I think that makes it worse, because you’re telling them what they should be doing. But if they haven’t got the ability to then do it, you’re in danger of making them feel quite bad about themselves, and then they end up with a guilt complex. It’s helping people to achieve it rather than telling them (12: Mother)

Respondents said it was important for people to trust, and also respect, the source of the information given. They wanted to know that professionals delivering messages about active living were speaking from personal experience, for the advice to have credibility. Information needed to be easy to understand and convey significance, for people to have confidence and be likely to respond. Respondents also suggested that the format and mode of message delivery should take into account socio‐cultural behaviour and preference.

…[For Asian families you need to] go to the home like the way the midwife comes to the home, talks to women, at that time people may accept it, I think so. Otherwise our Asian people don’t bother with other things [and disregard healthy living advice] (2: Mother)

Both parents needed to be engaged and included in discussions and information sharing around child and family physical activity.

Men have just as much role nowadays with bringing up children as women do and yeah, I suppose they need to put the information across to both parents, not just mother and baby sessions, you know, so they need to target men…if they like watching the football… (21: Mother)

Respondents distrusted advertising and were wary that the media misrepresented facts about physical activity. Within their localities, they favoured using community and social networks and encouraging word of mouth communication, to strengthen local connections alongside promoting community‐based physical activities. Parents from both types of neighbourhood expressed a need to connect and integrate with their neighbours and the wider community. They suggested community‐based activities as a way to achieve this. Some ideas concerned child‐focused neighbourhood events which did not appear to involve parents in activity. Other respondents suggested ways for communities to unite in physical activity by bringing parents together. Ideas included neighbourhood charity fundraising physical activities; community days out, bringing families together to be active; walk‐to‐school schemes to incorporate physical activity into daily routines and also reduce traffic volume and pollution; activities attached to community parenting classes and childcare provision; and community gardening areas to provide family physical activity and encourage healthy eating (see also Table 3):

…you’re also generating community spirit as well if people are interacting. I think that’s the best way (28: Father)

## 4 DISCUSSION

This study provides insights into factors influencing child and family‐based physical activity and includes recommendations from parents on ways to improve opportunities for family‐based physical activity in the community. Their suggestions have been summarized in Table 4. We found that parents from deprived neighbourhoods are more likely to be underactive because of a complex web of community, economic, social and personal factors which block motivation and opportunity for physical activity. Respondents said both parents need to be involved in designing interventions to support greater physical activity and healthy behaviours if they are to be relevant and achievable in individuals’ lives. Few physical activity‐based interventions have targeted both mothers and fathers of young children,^15^, ^49^ or have focused on facilitators and barriers to parent and child physical activity in the community.^50^ Engaging users to identify, design and deliver interventions is recommended to remedy current unhealthy lifestyles and lack of engagement with health messages.^51^, ^52^

**Table 4:** Respondent suggestions to encourage child and family physical activity

Information sharing
Targeted mailings about local activities issued before school holidays Location‐specific advertising of activities, focusing on community relevance Awareness raising by health professionals through pre‐ to post‐natal networks Link with formal and informal parent and toddler networks
Extending existing services
Physical activity sessions at community parenting classes Route cards for local cycle paths and walks Childcare at adult activity sessions Improve lighting and cleanliness of cycle routes and walking paths More activity clubs in deprived areas Make use of school and community facilities after school hours
Community activities
Charity fundraising activities: cycling/walking/jogging Active family days out Walk‐to‐school schemes Community garden areas Youth clubs and activities for teenagers

We identified barriers to family‐based physical activity which align with the levels described in Bronfenbrenner's socio‐ecological model.^31^, ^40^ We also found that some issues cross the ecological levels: work patterns and income (exosystem) affect individual isolation and well‐being (microsystem); and the quality of built environments and neighbourhood facilities (mesosystem) was associated with socio‐economic characteristics (macrosystem).^41^, ^53^, ^54^ While higher income and better quality environment enabled more physical activity, perceptions and attitudes were common between families in deprived and also affluent neighbourhoods. These findings are consistent with previous studies which report pressures at the exosystem and mesosystem level of work and family life encountered by parents when trying to maintain physical activity.(6, 36, 37, 38, 39, 40, 41, 42, 43, 44, 45, 53) In particular, we found the dynamic interplay between ecological levels reduced opportunities for physical activity for those parents living in deprived neighbourhood. This included the cost of activities^36^, ^37^ which limited access to transport and lack of time to accommodate physical activity^36^, ^37^, ^38^ because of irregular work patterns. Irregular and variable work patterns have been shaped by the expansion of a de‐regulated labour market resulting in a societal change across the macrosystem which has affected lower socio‐economic groups’ disproportionately.^55^ Indeed, parents in less affluent areas perceived physical activity as a luxury,^54^, ^56^ and therefore, it was not a regular occurrence. In contrast, parents living in affluent areas reported working mostly regular hours during the week and were therefore able to maintain some form of regular physical activity outside the home.

Obesity and other risks to adult health associated with variable work patterns, as well as the disruptive effect on the mesosystems such as family routines, have been documented.^57^, ^58^, ^59^, ^60^ Our findings reinforce evidence that shift work can limit involvement in regular extra‐curricular activities and general socialization in the community for families with young children in particular when both parents were working opposite shifts. We found one parent cannot always facilitate physical activity without partner or family support, which reduced child activity.^56^, ^61^ We now need to understand how the wider socio‐economic context of parents’ variable employment patterns impacts on child health and physical activity at the microsystem level and devise targeted interventions at one or more socio‐ecological levels to facilitate improved interactions between microsystem, mesosystem and exosystem factors.

Parents were not always aware of physical activities for families in their local area (ie mesosystem). Interventions targeted at the exosystem level could direct health professionals to provide information on available local opportunities for physical activity and the health benefits of being active for parents with infants and toddlers. Indeed, parents may be more inclined to enable family physical activity if they were aware of the importance for early motor development and future child health at the individual microsystem level.^62^ However, Bronfenbrenner's ecological theory^42^, ^43^ would suggest that changes in the mesosystem or exosystem are unlikely to be successful in isolation. Nutbeam^63^ states that providing information alone is unlikely to change behaviour. Acceptance of public health messages is determined by the way in which individuals interpret and internalize such messages. This can be mediated by microsystem factors such as individual autonomy and the ability to utilize this message, governed by contextual factors such as perception of lifestyle, level of education, disposable income, the physical environment and available support at the mesosystem and exosystem level.^28^, ^64^ Health‐orientated messages delivered at different ecological levels need to be simple, clear and consistent to be effective^65^; explain the importance of physical activity, convey the type of physical activity people should do, and ways they can be physically active.^66^ Awareness raising messages can also fail to reach the target audience due to timing of interventions, and lack of publicity and poor practitioner support.^67^ Parents in our study suggested ‘help not tell’ messages covering ‘why’, ‘how’ and ‘where’ information about physical activity. They suggested tailoring information for fathers and minority ethnic groups and using community and social networks for dissemination.

Participants in our study who were physically active outside the home often mentioned enjoyment and general well‐being for the whole family as a motivation. People who perceive a purpose in physical activity are more likely to take part and enjoy it at the individual microsystem level.^68^ Respondents who advocated community activities (charity bike rides, walking to school schemes) at the mesosystem level wanted to know their neighbours. This was particularly evident among respondents in deprived neighbourhoods who saw physical activity as a means of community engagement and social interaction to improve community cohesion. Thus, they advocated opportunities to engage in purposeful physical activity that accrued benefits above and beyond the individual. Research shows that community engagement can encourage physical activity and increase perceived social cohesion.^69^, ^70^, ^71^ Social interaction is also a motivator for family physical activity^68^, ^72^, ^73^ and may increase participation in community physical activity.^72^ Our findings identified that physical activity may also foster social interaction and this message may provide the purposeful motivator for individuals who feel disengaged and isolated.

Our study highlighted problems at all levels of the socio‐ecological model (ie microsystem, mesosystem and exosystem) preventing parents from deprived neighbourhoods from undertaking physical activity with their children. In common with people generally, they preferred to visit quality green spaces, the sea or leisure facilities, despite needing to travel a distance.^74^, ^75^, ^76^ Compared to those in affluent neighbourhoods, facilities local to them were inadequate, pushing them further afield in search of safe, accessible facilities. However, they physically struggled to leave their homes when the other parent was working, often lacked transport and were unable to meet the costs. Motivation and choice were consequently diminished. Frequency of outdoor activity reduces with distance required to travel to suitable locations, while access to personal transport usually increases physical activity in a choice of locations that offer varied experience.^77^

People with access to green spaces often exhibit better health‐related outcomes.^78^ Subsidized access to travel and venues that facilitate physical activity and more places for organized activities could help reduce these challenges at the mesosystem level. Improvements to neighbourhood facilities such as parks, paths and cycle ways could also address the reported negative perceptions.^79^, ^80^, ^81^ Physical activity generally increases in areas with more sports and recreational facilities, and attractive parks and cafes.^82^, ^83^, ^84^ Free outdoor recreation that is safe and attractive makes it visible within a community, positively influencing social and cultural attitudes towards physical activity.^83^ Such a change at the exosystemic level could bring about macro‐level changes which normalize physical activity and improve community cohesion, which was a priority for parents in this study. Public spaces near to bus stations and shopping areas could encourage both parent and child activity. Well‐designed neighbourhoods also enable more physical activity.^75^

Initiatives at the exosystem and macrosystem level^40^ require political commitment and resources to make societal changes impacting on health inequality.^85^, ^86^ In the short term, interventions that can promote physical activity in communities include improvements in active transport, housing location, urban design and neighbourhood safety.^26^, ^41^, ^87^, ^88^ Most successful multi‐component interventions can be seen to echo Bronfenbrenner's socio‐ecological theory where they are delivered alongside and supported by social marketing programmes that have raised awareness about the positive benefits of making healthy behaviour change.^89^

### 4.1 Strengths and limitations

The size of this study sample, which also included mothers and fathers, is a strength of this study. Respondents came from diverse socio‐economic and cultural groups and provided a rich and widespread of views. Where both parents were present, we were able to obtain varying perspectives on the topic. Parents either confirmed or contradicted a point or added detail to each other's responses. However, we acknowledge that the presence of the partner could have negatively influenced some responses where respondents felt unable to speak freely. There was a possibility of participant bias as mothers had already agreed to participate in a birth cohort study so may have been more motivated towards healthy behaviours compared to the general population. However, views appeared generally consistent within socio‐economic groups, suggesting the responses concerned issues shared by those populations.

## 5 CONCLUSION

Parents from deprived neighbourhoods experience a range of community, social, economic and personal barriers which interact to limit their ability to be physically active with their children. To increase knowledge and opportunity relating to physical activity, they suggested ‘help not tell’ messages covering ‘why’, ‘how’ and ‘where’ information, tailored for audiences including fathers and minority ethnic groups. They saw physical activity as a means of community engagement and social interaction and advocated local activities for children and families. Engaging parents and communities in identifying, designing and delivering interventions may help support increased physical activity which are relevant and achievable in individuals’ lives.

## CONFLICT OF INTEREST

The authors declare they have no actual or potential competing financial interests.

## AUTHORS’ CONTRIBUTIONS

RAL and SB designed the ‘Growing Up in Wales’ birth cohort study. AK, RAH and KM were involved in the recruitment of participants to this birth cohort study. AK conceived the qualitative research concept and study design and sampled participants from the birth cohort study for interview. AK developed the interview schedule with advice from SB and RAH. AK conducted all the interviews. AK, RL and EM analysed and interpreted the qualitative interview data and drew up a draft document of themes. FR offered guidance on qualitative methodology. AK drafted the manuscript with support from BAE, and all authors read and approved the final manuscript.

## DATA AVAILABILITY STATEMENTS

The data that support the findings of this study are available from the corresponding author upon reasonable request.
